# Procyanidins Negatively Affect the Activity of the Phosphatases of Regenerating Liver

**DOI:** 10.1371/journal.pone.0134336

**Published:** 2015-07-30

**Authors:** Sven Stadlbauer, Pablo Rios, Ken Ohmori, Keisuke Suzuki, Maja Köhn

**Affiliations:** 1 European Molecular Biology Laboratory, Genome Biology Unit, Meyerhofstrasse 1, 69117, Heidelberg, Germany; 2 Tokyo Institute of Technology, Department of Chemistry, O-okayama, Meguro-ku, Tokyo, 152–8551, Japan; Faculty of Medicine, BELGIUM

## Abstract

Natural polyphenols like oligomeric catechins (procyanidins) derived from green tea and herbal medicines are interesting compounds for pharmaceutical research due to their ability to protect against carcinogenesis in animal models. It is nevertheless still unclear how intracellular pathways are modulated by polyphenols. Monomeric polyphenols were shown to affect the activity of some protein phosphatases (PPs). The three phosphatases of regenerating liver (PRLs) are close relatives and promising therapeutic targets in cancer. In the present study we show that several procyanidins inhibit the activity of all three members of the PRL family in the low micromolar range, whereas monomeric epicatechins show weak inhibitory activity. Increasing the number of catechin units in procyanidins to more than three does not further enhance the potency. Remarkably, the tested procyanidins showed selectivity in vitro when compared to other PPs, and over 10-fold selectivity toward PRL-1 over PRL-2 and PRL-3. As PRL overexpression induces cell migration compared to control cells, the effect of procyanidins on this phenotype was studied. Treatment with procyanidin C2 led to a decrease in cell migration of PRL-1- and PRL-3-overexpressing cells, suggesting the compound-dependent inhibition of PRL-promoted cell migration. Treatment with procyanidin B3 led to selective suppression of PRL-1 overexpressing cells, thereby corroborating the selectivity toward PRL-1- over PRL-3 in vitro. Together, our results show that procyanidins negatively affect PRL activity, suggesting that PRLs could be targets in the polypharmacology of natural polyphenols. Furthermore, they are interesting candidates for the development of PRL-1 inhibitors due to their low cellular toxicity and the selectivity within the PRL family.

## Introduction

**Fig 1:**
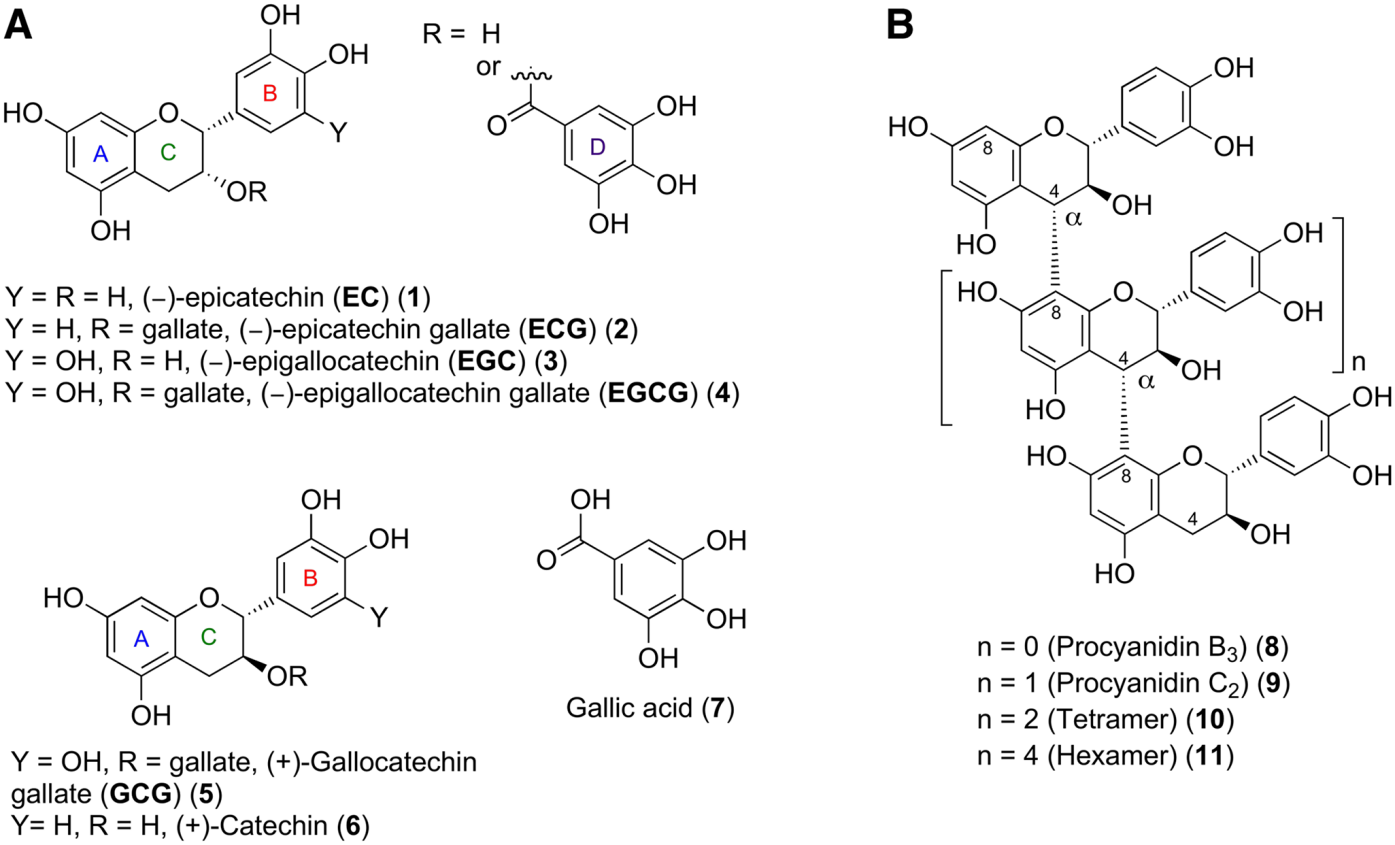
Structures of catechin-type polyphenols. (A) monomeric polyphenols and (B) oligomeric catechins with B-type (4→8) linkage (procyanidins).

Natural polyphenols are subject to increasing interest due to their interesting pharmacological activities [1–4]. Especially catechin-class polpyhenols (see Fig 1A) such as the green tea polyphenols (–)-epigallocatechin (EGC) and its 3-*O*-gallate (EGCG) have attracted strong attention due to their health benefits. Less abundant epicatechins contained in green tea are (–)-epicatechin and (–)-epicatechin-3-*O*-gallate (ECG). Research in the past decade has shown that those tea polyphenols are able to protect against tumor initiation and promotion in animal models [5], however, the understanding of intracellular pathways modulated by these compounds is still incomplete. EGCG, the most abundant green tea polyphenol, was found to induce cell cycle arrest and apoptosis in many cancer cells without affecting normal cells [6]. Their antitumor activity is supposed to be exerted by targeting multiple pathways involved in cancer progression, such as the NF-kappaB, MAPKs, EGFR and IGF-I mediated pathways [7]. More recently it was shown that EGCG functionally antagonizes androgen action at multiple levels in prostate cancer, resulting in inhibition of prostate carcinoma cell growth, thus making it a promising chemotherapeutic agent against hormone-refractory prostate cancer [8].

Besides monomeric catechins, nature produces a huge “catechin library” consisting of oligomers with varying degree of oligomerization as well as different connection patterns, such as B-type linkages [9,10] as shown in Fig 1B. These condensed tannins or proanthocyanidins are ubiquitously spread in the plant kingdom and are known to exhibit a wide range of antiviral, antibacterial, and antitumor activities [11–14]. These biologically active structures are composed of repeating units of catechins or epicatechins and can form homo and heterooligomers [15]. Despite a potential rich source of compounds of biological and pharmacological relevance, progress in this field was hampered due to (i) the potential broad, nonspecific binding to different proteins [9] and (ii) the difficulties in obtaining pure samples of individual compounds. Therefore, previous biological studies were conducted mostly with mixtures of species. This, in turn, made it difficult to study mechanisms of action, which are still largely unknown [16]. However, in recent years others and we have developed several synthetic strategies to access catechin-type polyphenols [17–19] as well as their oligomers in sufficient amounts [20–24], enabling now to study biological effects of the pure compounds.

In one study, pure defined procyanidins (dimeric to undecameric (–)-epicatechin) were observed to induce cell cycle arrest in breast cancer cell lines, while showing a strong increase of cytotoxicity with increasing chain length of the oligomer [23]. A study with pure prodelphinidin B3 and C2 showed that they inhibit the growth of prostate cancer cells through cell cycle arrest and caspase-3 activation [25]. Another study with monomeric to trimeric catechins showed that procyanidin C2 (trimer) stimulated TNF-alpha sectretion, NO production and NF-kappaB-dependent gene expression induced by interferon gamma in RAW 264.7 macrophages [26].

Amongst the known affected proteins of polyphenols are protein phosphatases (PPs), although detailed studies are still rare. Recently, Erdődi and coworkers reported that EGCG is a strong inhibitor of protein phosphatase-1 (PP1) while showing a ten-fold lower activity toward the close homologue PP2A, thus making it a partly selective inhibitor [27]. For procyanidins such studies are missing for both mixtures of procyanidins as well as pure compounds. PPs are interesting targets as they are involved in several pathogenic mechanisms leading, for example, to cancer. Two major PP families are the protein serine/threonine phosphatases (PSTPs), containing PP1 and PP2A, and the protein tyrosine phosphatases (PTPs). These families are evolutionary distinct and were classified originally according to their substrate specificity, but the PTP family contains members, so-called dual specificity phosphatases (DSP), that can dephosphorylate phosphoserine or threonine residues and also non-protein substrates [28]. The phosphatases of regenerating liver (PRLs) belong to the DSPs. The three PRLs PRL-1, PRL-2 and PRL-3 are relatively small proteins of ~22 kDa with at least 75% amino acid sequence similarity [29,30]. PRLs are being evaluated as cancer biomarkers and promising therapeutic targets in cancer [31–33]. PRL-3 promotes cancer metastasis and enhances proliferation in many primary cancers, and promotes cell migration in cell culture. In addition, its high abundance correlates with poor prognoses for cancer patients [31]. PRL-1 and PRL-2 are also overexpressed in several cancers and their overexpression is correlated with tumor progression [34–36]. The underlying molecular mechanisms are, however, not well understood and substrates are not established [33]. Among natural potential inhibitors for PRL-3 are the flavonols myricetin and gossypin, another subclass of polyphenols, showing moderate phosphatase inhibition *in vitro* [37].

Here, we aimed to examine whether the inhibition of PPs by specific polyphenols, such as epi-type catechins and their 3-*O*-gallates (Fig 1A) as well as (+)-catechin constituted oligomers (procyanidins) (Fig 1B) could contribute to the pharmacological activity of foods and beverages such as green tea. Our focus was on PRL-3 due to its role in cancer and the reported antitumor activities of polyphenols. Whereas naturally occurring monomeric polyphenols inhibited PRL-3 only weakly, oligomerization appeared to increase the potency starting from the trimeric compound. The oligomeric polyphenols also inhibited other PPs, both PTPs and PSTPs, with varying potencies. Since the trimer procyanidin C2 showed one of the highest inhibitory potencies against PRL-3 *in vitro*, we performed studies in HEK cells to examine its cytotoxicity and ability to suppress cell migration via inhibition of PRL-3. We observed cytotoxicity of procyanidin C2 only at high micromolar concentrations. The compound itself induced cell migration of control cells at a non-cytotoxic concentration, and reduced the PRL-3-induced cell migration to the level of the compound-treated control cells. These results show that this compound has complex cellular effects, including the inhibition of PRL-3 and -1-induced cell migration. Among the oligomeric catechins procyanidin B3 showed remarkable selectivity toward PRL-1 in vitro, which was mirrored in cells by procyanidin B3-mediated reduction of migration speed of PRL-1- but not of PRL-3-overexpressing cells. Unlike procyanidin C2, procyanidin B3 did not induce cell migration in control cells, demonstrating a clear activity on PRL-1. Together, our results indicate that the inhibition of several phosphatases from different phosphatase families by these polyphenols is likely to contribute to the pharmacological activity of these compounds, including the inhibition of the cancer-promoting PRLs.

## Results

### Effect of polyphenols on the *in vitro* activity of protein phosphatases

**Fig 2:**
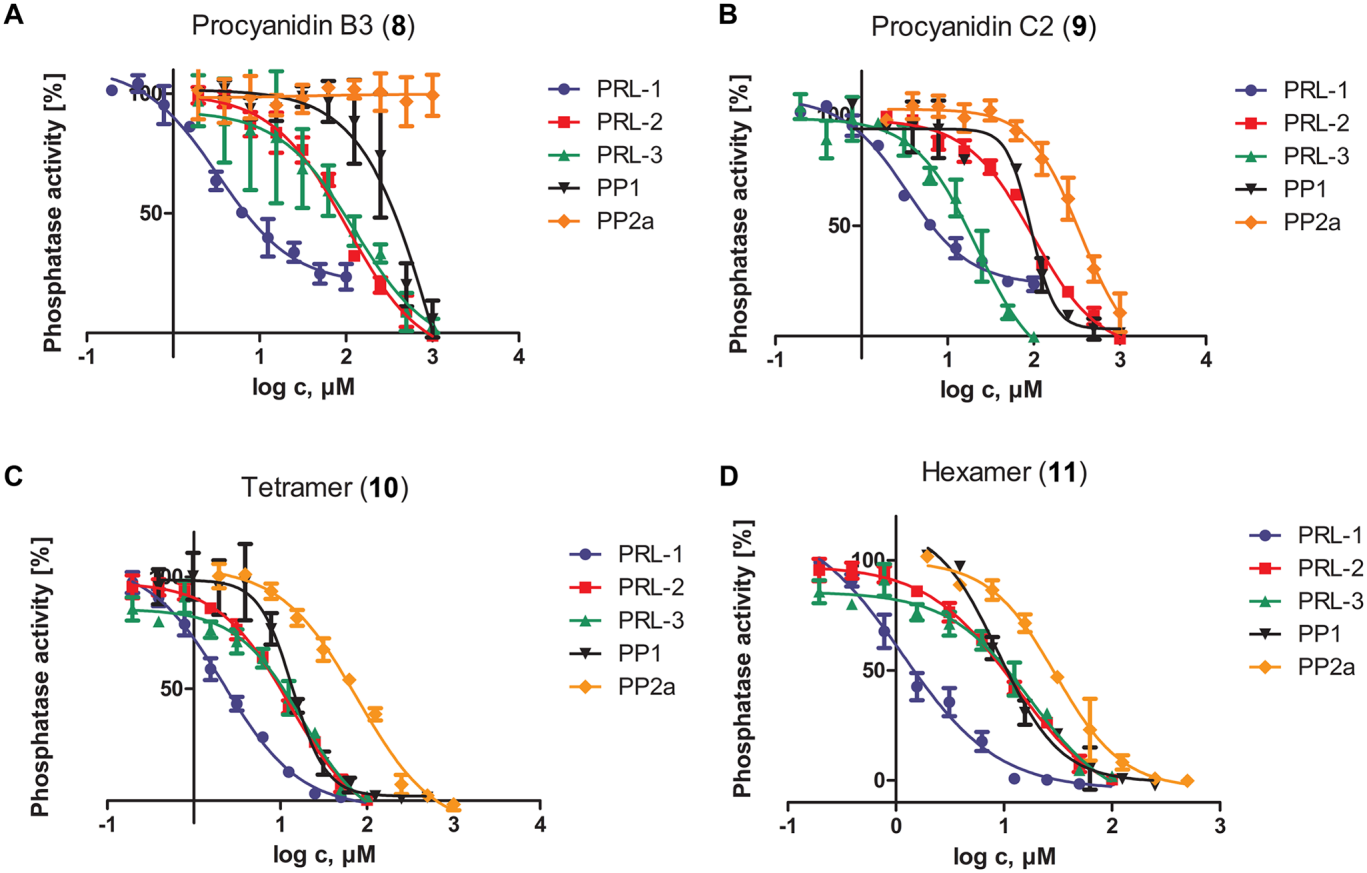
Effect of polyphenolic compounds 8 (A), 9 (B), 10 (C) and 11 (D) on the phosphatase activity of PRL-1, PRL-2, PRL-3, PP1, and PP2A. Proteins were incubated for 30 min with 0–1000 μM of the corresponding procyandin **8**–**11**. Phosphatase activity was measured in the presence of DiFMUP at 25°C using the concentration corresponding to the K_M_ of each protein. The K_M_ for PRLs was determined separately (PRL-3 = 21 μM, PRL-2 and PRL-1 = 24 μM). The K_M_ for PP1 (91 μM) and for PP2A (100 μM) was taken from the literature [38,39]. Protein concentrations are 50 nM for all PRLs and 2 mU for PP1 and 0.05 U for PP2A (see the Experimental Procedures). Phosphatase activity in the absence of inhibitors was set as 100%. Data represent means ± standard errors of the mean (n = 3–5).

Biochemical phosphatase activity measurements were carried out in the presence of different concentrations of various polyphenols, compounds **1**–**11** (Fig 1), using recombinant PRL-3 and 6,8-difluoro-4-methylumbelliferyl phosphate (DiFMUP) as a fluorogenic substrate (see the experimental procedures). Results are shown in Table 1 and Fig 2. We observed that the monomeric epicatechins EGC and EC inhibit PRL-3 only weakly, with a slightly better activity of EGC, which bears three hydroxyl groups at the B-ring. This is in agreement with the findings of He et al. who observed that the hydroxyl groups at position 4’ (at the B-ring) and 7 (at the A-ring) (see numbering scheme in Fig 1) are important for exhibiting inhibitor activity toward PRL-3 [37]. An about two-fold increase in inhibition was observed for compounds **2** and **4** bearing a gallate ester at the 3-position of the molecule compared to **1** and **3**, respectively. However, gallic acid (**7**) itself did not show any inhibition up to 1000 μM. This indicates that the gallate moiety in combination with the flavan skeleton is beneficial for activity. In general, as a basic structure activity-relationship the following trend was observed: a higher number of hydroxyl groups at the B-ring increased the activity slightly. However, attachment of a gallate ester (D-ring) at position 3 of the flavan skeleton significantly increased the inhibitor activity. Therefore, combination of a pyrogallol ring as the B-ring and a gallate ester at position 3, exhibited the highest activity in these series. The influence of the stereochemistry at C(2)–C(3) was studied by using GCG (the *trans*-epimer of EGCG). The results indicated that both epimers suppress the activity of PRL-3 similarly. The same observation was made by using (+)-catechin (**6**, IC_50_ = 456 μM vs. 514 μM for EC(**1**)). Thus the C(2)–C(3) stereochemistry does not have significant influence on the inhibitor activity of the corresponding flavanol on PRL-3.

**Table 1:** IC_50_ (= K_i_) values of polyphenolic compounds 1–11 for the inhibition of protein phosphatases. IC_50_ values are mean ± standard error of the mean (*n* = 3–5).

Compound	IC_50_ (K_i_) [μM]				
	PRL-3	PRL-2	PRL-1	PP1	PP2A
EC (**1**)	514 ± 68.2	n.d.	n.d.	n.d.	n.d.
ECG (**2**)	181 ± 13.6	n.d.	n.d.	n.d.	n.d.
EGC (**3**)	285 ± 3.7	n.d.	n.d.	n.d.	n.d.
EGCG (**4**)	121 ± 5.7	n.d.	n.d.	n.d.	n.d.
GCG (**5**)	113 ± 10.7	n.d.	n.d.	n.d.	n.d.
(+)-Catechin (**6**)	456 ± 65.6	n.d.	158 ± 1.2	n.d.	n.d.
Gallic acid (**7**)	> 1000	n.d.	n.d.	n.d.	n.d.
Procyanidin B3 (**8**)	127 ± 1.5	103 ± 1.1	3.3 ± 1.2	383 ± 43	>1000
Procyanidin C2 (**9**)	18.8 ± 4.5	15.2 ± 1.4	1.8 ± 1.1	88 ± 13.7	213 ± 72
Tetramer (**10**)	16.3 ± 2.3	13.4 ± 2.5	2.2 ± 1.1	13.8 ± 2.9	77.9 ± 10.7
Hexamer (**11**)	12.5 ± 1.8	10.0 ± 0.4	1.2 ± 0.6	7.0 ± 2.3	32.5 ± 4.8

Next we studied the procyanidins (compounds **8**–**11**), which are derived from oligomerization of (+)-catechin (**6**). Noteworthy, in contrast to the whole (epi)catechin series (compounds **1**–**7**) the water solubility of these oligomers is very high, therefore, DMSO could be completely excluded from the assay buffer system. The results (Fig 2A–2D and Table 1) showed a strong increase of inhibitor activity with increasing chain length of the oligomer up to procyanidin C2 (trimer, **9**). Further extension of the oligomeric chain to tetramer and hexamer did not lead to any significant increase in phosphatase inhibition. Since oligomers **8**–**11** were more potent than the monomeric ones, they were further studied with regard to their activity toward the other two members of the PRL family. For PRL-2 the inhibition was in the same range as for PRL-3, giving IC_50_ values between 103 μM (**8**) and 10 μM (**11**). Statistical analysis of the IC_50_ values using unpaired t-test with Welsh correction proved that there is no significant difference between the inhibitory potency of the oligomers, except for **8**, toward PRL-3 and PRL-2. In contrast, all procyanidins suppressed PRL-1 activity more potently as can be seen for example for procyanidin B3 (**8**), which showed an IC_50_ value of 3.3 μM for PRL-1 compared to 127 μM for PRL-3. Thus, it showed a 38-fold higher activity toward PRL-1 compared to PRL-3, which is the largest selectivity range among the procyanidin series. Unpaired t-testing proved a statistically significant difference for compound **8** between the inhibition of PRL-3 and PRL-1. In contrast, for trimer **9** only a weak significant difference was observed. For the tetrameric and hexameric compounds, similar IC_50_ values around 1.5 μM were observed. To verify whether this strong inhibition is already caused by the monomeric building block (+)-catechin (**6**), we checked its inhibitor potency as well. The resulting IC_50_ value of 158 μM toward PRL-1 is about three times lower than for PRL-3, but 50-fold higher than the IC_50_ values obtained for the oligomers. In conclusion, these data show that in vitro, procyanidins inhibit PRL-1 stronger than the other two PRLs.

Kiss et al. showed that the activity of protein phosphatase-1 is potently inhibited by EGCG and 1,2,3,4,6-penta-*O*-galloyl-beta-d-glucose (PGG) exhibiting IC_50_ values of 1.35 and 0.4 μM, respectively [27]. In contrast, the structural homologue PP2A was less potently inhibited, thus showing a clear selectivity toward PP1. Therefore, we studied the most potent compounds **8**–**11** toward their suppression of PP1 and PP2A activity (PP1 and PP2A originate from different sources and were used in different concentrations, therefore the IC_50_/K_i_ comparison needs to be viewed with caution, see the Experimental Procedures and below). The phosphatase activity of both proteins was examined using again the fluorogenic substrate DiFMUP [38,39], which was found to provide lower IC_50_ values than the pNPP assay and thus is closer to the data obtained from the very sensitive phosphorylase assay [38]. BSA, which is usually supplemented to the assay buffer for stabilizing the PPs was however excluded, as BSA is known to form complexes with polyphenolic compounds [40–42] and thus would decrease the concentrations of the inhibitor in the assay. The results are summarized in Fig 2A–2D and Table 1. The data indicate a similar trend for PP1 as for the PRLs: that the inhibitor potency increased with increasing molecular weight of the oligomers until the tetramer (**10**). The potency of hexamer **11** increased only about two-fold compared to tetramer **10**, with an IC_50_ value of 7 μM. In contrast, the phosphatase activity of PP2A was suppressed to a much lesser extent with weak (for trimer **9** and hexamer **11**) and strong statistical significance (dimer **8** and tetramer **10**). Thus our results corroborate the data reported previously [27].

**Fig 3:**
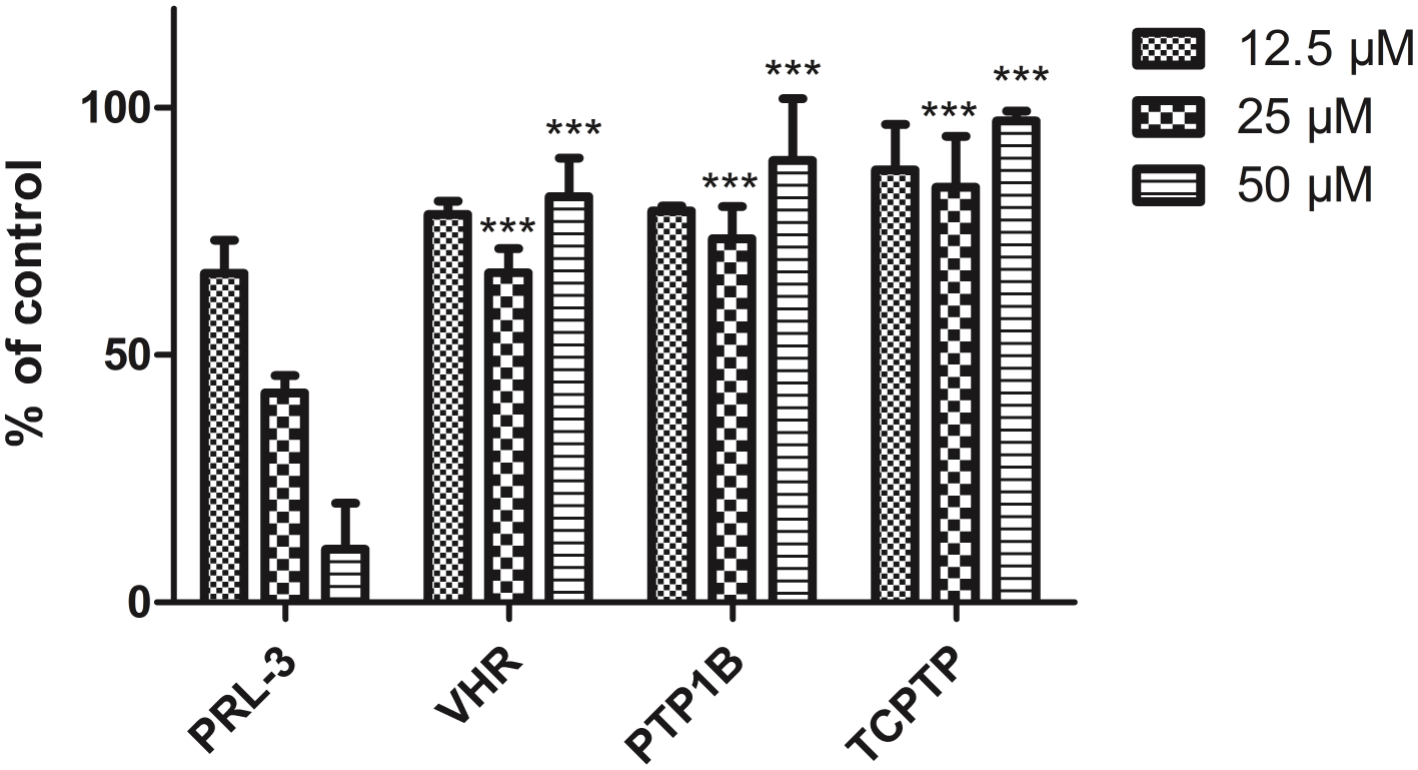
Effect of procyanidin C2 (9) on the phosphatase activity of VHR, PTP1B and TCPTP using DiFMUP as a substrate [44]. Proteins were incubated with compound **9** for 30 min., phosphatase activity measurements were performed using 20 μM DiFMUP. Phosphatase activity in the absence of compound **9** was set as 100%. Data represent means ± standard errors of the mean (n = 3).

As an extension of our studies we included PTP1B, VHR and TCPTB as examples for phosphotyrosine specific phosphatases in our activity studies, because tea extracts were reported to inhibit PTP1B [43]. For comparison with PRL-3, these proteins were used in the same concentration (50 nM) as PRL-3 and DiFMUP was also used as a substrate in 20 μM concentration [44]. The enzymes were incubated with three different concentrations of procyanidin C2 (**9**) for 30 min before the measurement. The data obtained (Fig 3), show that at 12.5 μM, phosphatase activity was reduced to 75–85% compared to the control, but there was no significant difference in activity suppression between PRL-3 and the other phosphatases. However, at 25 μM and 50 μM the activity of PRL-3 was inhibited in a concentration dependent manner whereas the three tested PTPs were not, showing that the compound is selective toward PRL-3 over the other three phosphatases. These results show that procyanidin C2 does not randomly inhibit any PTP, but is active toward the PRLs.

**Fig 4:**
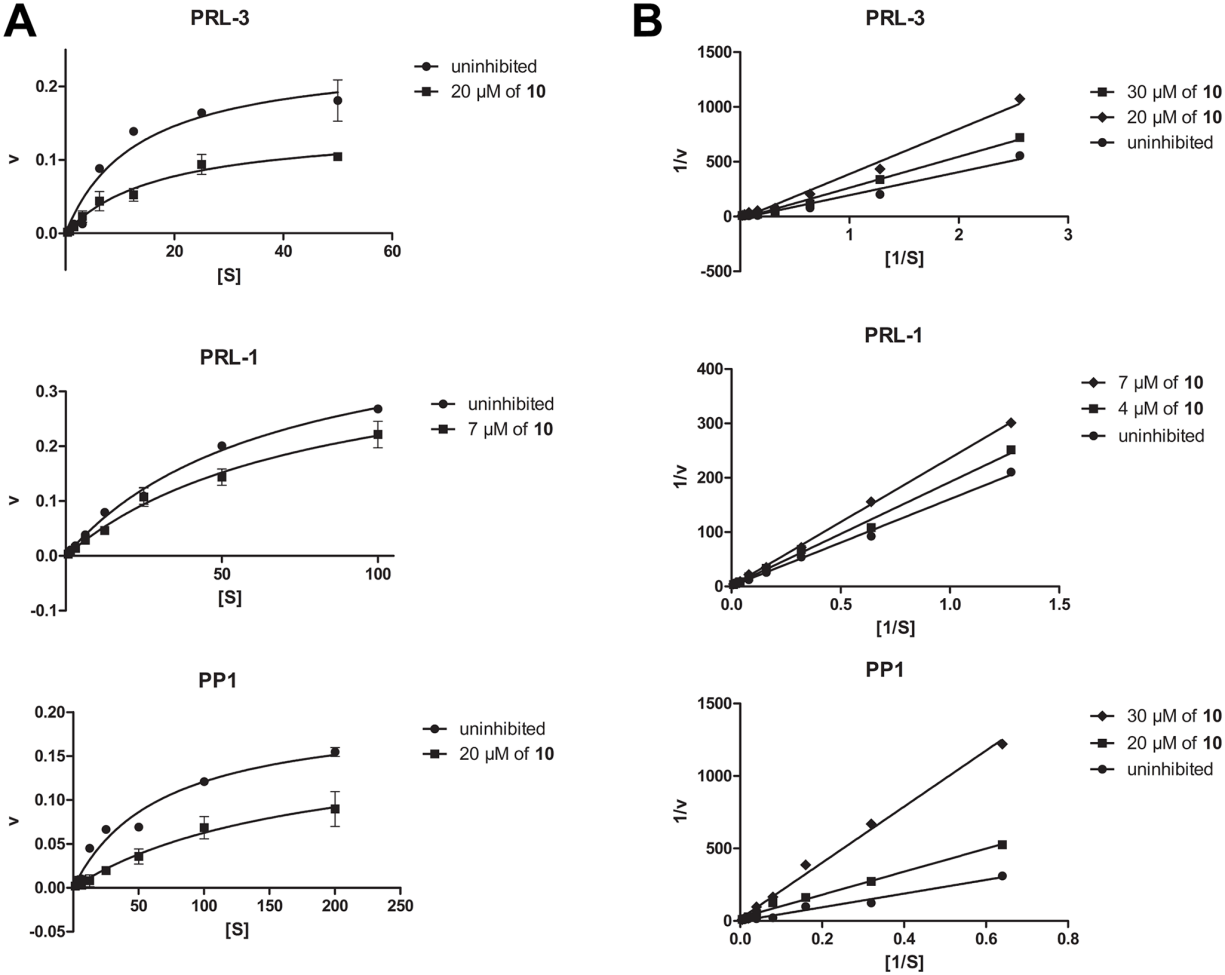
Analysis of the type of inhibition for compound 10. Michaelis-Menten plot (A) and Lineweaver-Burk plot (B) of **10** inhibiting DiFMUP dephosphorylation by PRL-1, PRL-3 and PP1. Both plots show typical noncompetitive behavior for all phosphatases. Phosphatase activity in the absence of compound **10** was set as 100%. Data represent means ± standard errors of the mean (n = 3).

In order to understand how procyanidins bind to the phosphatases, we studied the type of inhibition. As a representative example tetramer **10** was chosen, because it inhibited all five phosphatases in the low micromolar range. Therefore, we performed detailed kinetic analyses on tetramer **10** inhibiting DiFMUP hydrolysis by PRL-1 and PRL-3 as representative examples for the PRLs and PP1 for the PPs. Plotting the initial velocities against different DiFMUP concentrations and analysis by Michaelis-Menten kinetics showed clearly a noncompetitive behavior for all three phosphatases examined (Fig 4A). These results were corroborated by a Lineweaver-Burk plot (see Fig 4B). In conclusion this experiment indicates that the catechin-class polyphenols such as tetramer **10** interact with the enzyme at a site different than the DiFMUP interaction site, and that this interaction is independent of binding of the substrate to the enzyme itself. The type of inhibition is an important parameter for calculating the K_i_ values that correspond to the IC_50_s. K_i_ values could enable a better comparison of the inhibitory potency of the compounds between the PRLs, PP1 and PP2A. However, for the classic mode of inhibition for a noncompetitive inhibitor the K_i_ is equal to the IC_50_ value, in this case not allowing for a PP concentration-independent comparison of the values.

### In-cell effects of polyphenols on PRL-3

**Fig 5:**
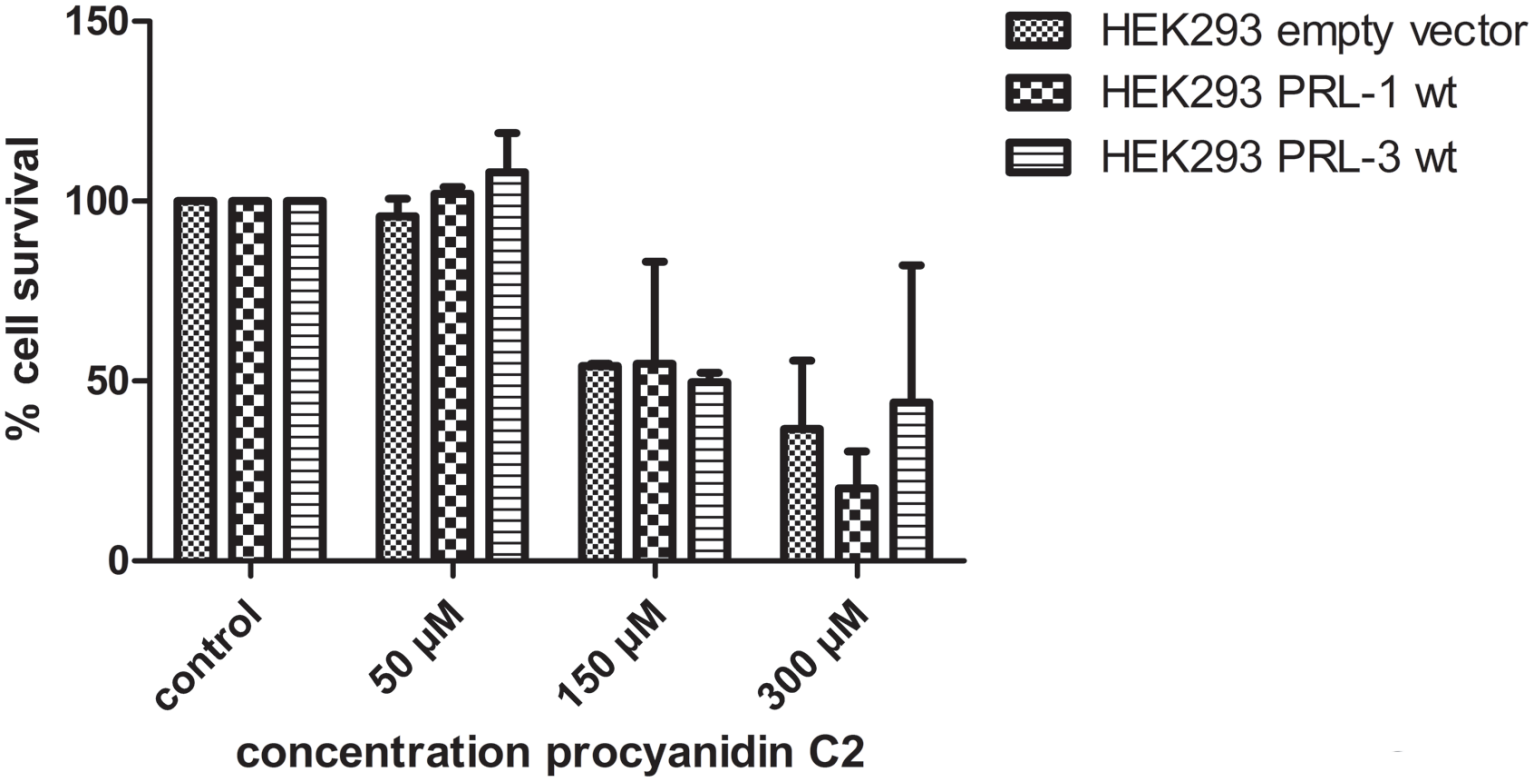
Cytotoxicity of procyanidin C2 (9) in HEK293 cells. Cells were treated with 50, 150 or 300 μM of **9** in the growth medium for 24 h. Cells were harvested, resuspended in PBS buffer and incubated with propidium iodide (1 mg/mL) for 5 min on ice. 10,000 events were set for the number of living cells. The number of living cells was divided by the number of total cells counted and given as percentage of cell survival. Data represent means ± standard errors of the mean (n = 3).

To study the effects of procyanidins on PRL-1 and PRL-3 in cells, the trimeric catechin (procyanidin C2, **9**) was chosen as a representative example due to its strong inhibitor activity in vitro toward both PRL-s and its smaller size compared to the higher oligomers, which could influence cell permeability. HEK293 cells stably overexpressing PRL-1 and PRL-3 were chosen as model cell line [45]. As a control cell line, HEK293 cells transfected with the empty vector were utilized (see the Experimental Procedures) [45]. To avoid false positive results due to cell death, first cell viability was studied in a concentration dependent manner by using flow cytometry. Therefore, cells were treated with different concentrations of compound **9** for 24 h, harvested, and then the ratio of living to dead cells was determined using propidium iodide (Fig 5). Treatment with 50 μM of compound **9** did not show any cytotoxic effects in either of the three cell lines, whereas concentrations of 150 and 300 μM generally reduced cell viability to about 50%.

**Fig 6:**
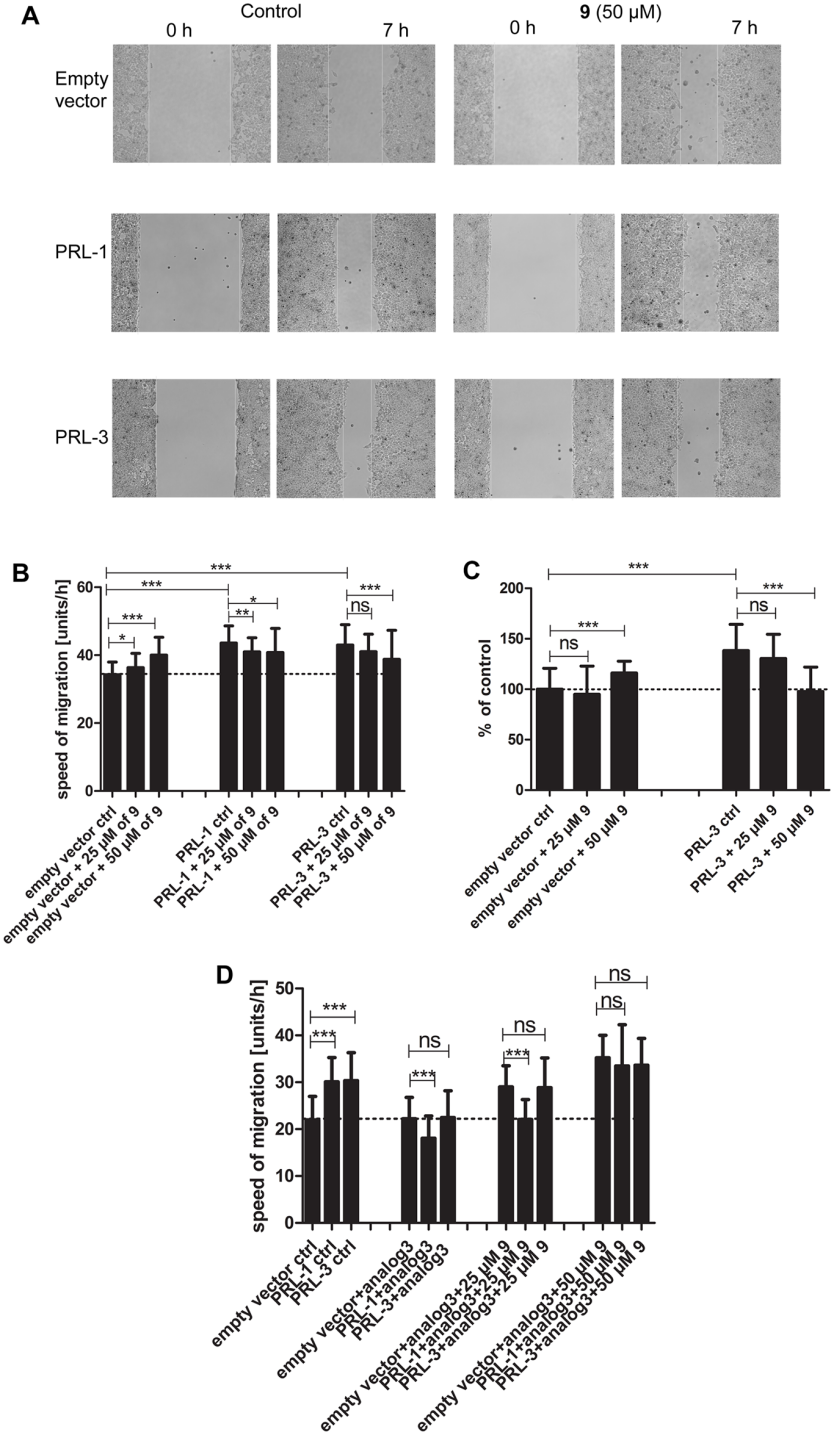
Wound healing abilities of PRL-1 or PRL-3 overexpressing or empty vector control HEK293 cell lines were analyzed in the presence of compound 9 or medium only. Cells were seeded at a concentration of 800,000 cells per insert and procyanidin C2 (**9**) was added at a concentration of 25 and 50 μM. A) Pictures of the wound gaps were taken at 0 h and 7 h for both conditions (with and without treatment). B) Quantification of the wound healing experiment. Speed of migration is shown for combined data from different replicates, as mean ± SD (n = 38–88). Two-sided t-tests with Welsh correction were performed for p < 0.05. ns = not significant;***: < 0.001; **: < 0.01; *: < 0.05. C) Effect of polyphenolic compounds in a transwell assay on the cell migration of HEK293 empty vector and PRL-3 overexpressing cell lines. Cells were seeded at a concentration of 100,000 cells per insert and procyanidin C2 (**9**) was added at a final concentration of 25 and 50 μM. Migration was allowed for 16 h before harvesting and staining with calcein AM for fluorescent readout. Mean ± SD (n = 60–72) is shown for combined data from different replicates. Two-sided t-tests with Welsh correction were performed for p < 0.05. ns = not significant. D) Quantification of wound healing experiment including blocking PRL-1 and PRL-3 with analog 3. Speed of migration is shown for combined data from different replicates, as mean ± SD (n = 63–82). Two-sided t-tests with Welsh correction were performed for p < 0.05.

To study if procyanidin C2 is able to inhibit PRL-1 and PRL-3 in cells, HEK293 cells were treated in a wound-healing assay (see the experimental procedures). The PRL-1 and PRL-3 stably overexpressing HEK293 cells were treated with 25 and 50 μM of **9**, since these concentrations did not cause any cytotoxic effects as mentioned above. As a control the empty vector cell line was again utilized. As expected [29,45], compared to the untreated empty vector control cell line the untreated PRL-3 and PRL-1 overexpressing cells showed a significantly higher migration due to PRL-1 and PRL-3 activity. Treatment with 25 μM of **9** slightly increased migration behavior in the empty vector cell line, and treatment with 50 μM of **9** significantly increased the number of migrating cells in the control cell line (see Fig 6A and 6B). In contrast, treatment of the PRL-1 cells with 25 μM of **9** showed a slight drop in cell migration, and for PRL-3 overexpressing cells the migration speed did not decrease significantly at 25 μM. When the concentration of **9** was increased to 50 μM, the cell migration speed of the PRL-1 overexpressing cell line did not change any further compared to 25 μM. At treatment with 50 μM **9**, the PRL-3 overexpressing cell line showed a significant decrease in cell migration compared to 25 μM of **9**. The migration speed was for all cell lines similar when treated with 50 μM of **9**. To further corroborate these findings we performed additionally a transwell assay as an alternative way to study cell migration [45]. Here again the untreated controls showed a significant increase in cell migration for the PRL-3 overexpressing cell line compared to the empty vector cell line. Treatment with 25 μM of **9** did not lead to a significant change in cell migration for neither the empty vector cell line nor the PRL-3 overexpressing cell line. In contrast, treatment with 50 μM of **9** lead to a significant increase in cell migration for the empty vector cell line and a significant decrease in migration for PRL-3 overexpressing cell line (see Fig 6C), with the speed dropping to the level of the untreated control cell line, corroborating the results from the wound healing assay.

To clearly show that the enhanced cell migration in PRL-overexpressing cells depended on the PRLs, and thus to support that the concentration-dependent inhibition of this phenotype by compound **9** was due to PRL inhibition, we applied the small molecule PRL inhibitor “analog 3” [45]. Treatment of the PRL-1 and PRL-3 overexpressing cells with 35 μM of analog 3 blocks PRL activity in the wound healing assay [45]. As can be seen in Fig 6D, the migration speed for PRL-3 overexpressing cells fell back to the baseline level of the empty vector cell line (dotted line) when treated with analog 3. The migration speed for PRL-1 overexpressing cells treated with analog 3 was a bit lower than the baseline. When adding compound **9** at a concentration of 25 μM, migration of the PRL-3 overexpressing cell line was the same as for the empty vector cell line. Migration of the PRL-1 overexpressing cell line was not fully restored to the same level as the empty vector cell line, but for 50 μM of **9** the migration speed of PRL-1 and PRL-3 overexpressing cell lines was exactly the same as for the empty vector cell line. While there is this small variation in the PRL-1-overexpressing cell lines, the trend is the same and overall we can conclude that procyanidin C2 (**9**) enhances cell migration in the tested range (25–50 μM) in a PRL-independent manner. Taken together, compound **9** appears to inhibit PRL-mediated cell migration for both PRL-1 and PRL-3 overexpressing cell lines in a dose-dependent manner. These results are in line with our in vitro assays. Nevertheless, in our cellular assay system we did not see the higher inhibitory potency of compound **9** toward PRL-1 over PRL-3 that we observed in vitro, which could be due to uptake issues [46] that even out the weak selectivity seen in vitro.

**Fig 7:**
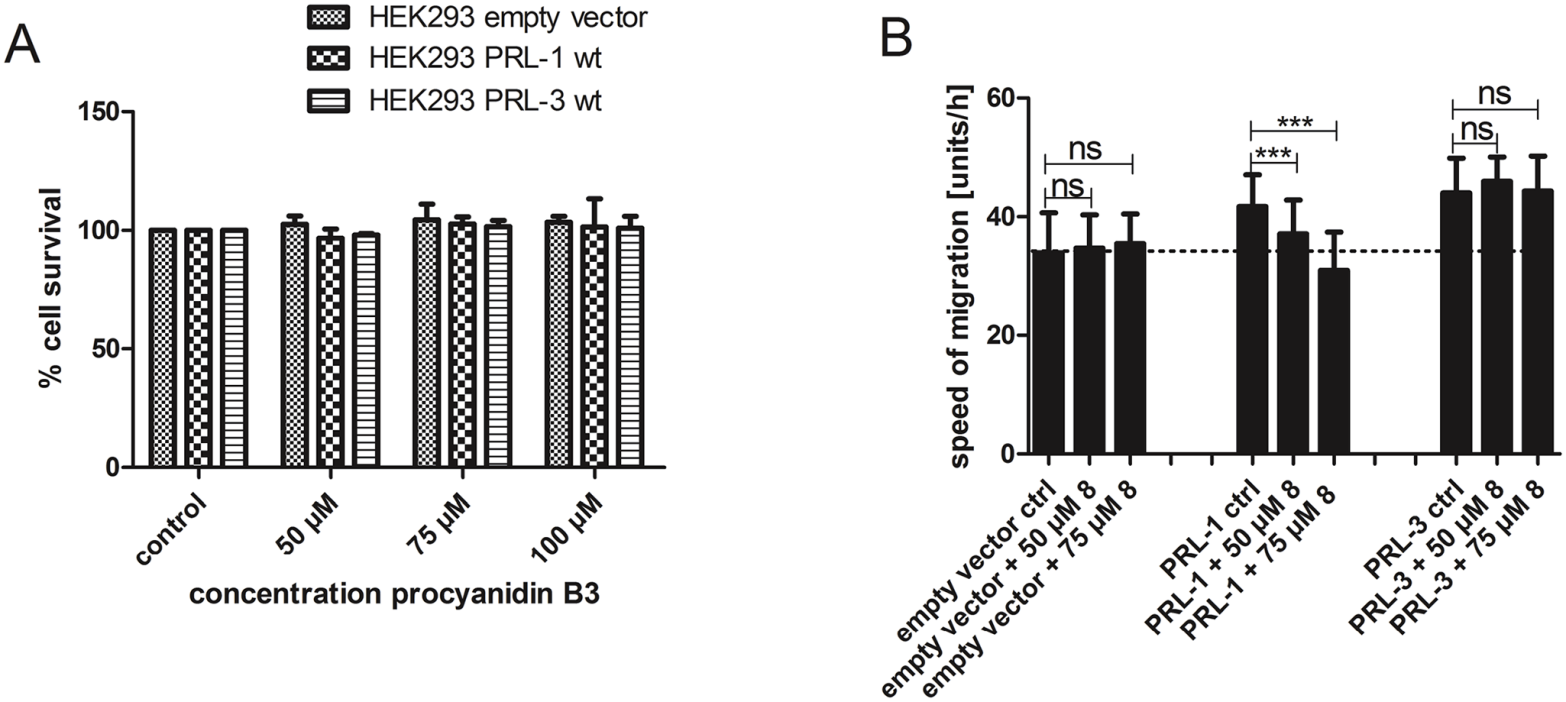
A) Cytotoxicity of procyanidin B3 (**8**) in HEK293 cells. Cells were treated with 50, 75 or 100 μM of **8** in the growth medium for 24 h. Cells were harvested, resuspended in PBS buffer and incubated with propidium iodide (1 mg/mL) for 5 min on ice. 10,000 events were set for the number of living cells. The number of living cells was divided by the number of total cells counted and given as percentage of cell survival. Data represent means ± standard errors of the mean (n = 3). B) Wound healing abilities of PRL-1 or PRL-3 overexpressing or empty vector control HEK293 cell lines were analyzed in the presence of compound **8** or medium only. Cells were seeded at a concentration of 800,000 cells per insert and procyanidin B3 (**8**) was added at a concentration of 50 and 75 μM. Speed of migration is shown for combined data from different replicates, as mean ± SD (n = 40–77). Two-sided t-tests with Welsh correction were performed for p < 0.05. ns = not significant;***: < 0.001; **: < 0.01; *: < 0.05.

Seeing the selectivity in cells that we observed in vitro would corroborate our findings that procyanidins affect PRL activity in cells. Therefore, we studied compound **8**, a catechin dimer with reported cell permeability [46] and with a much stronger significant difference in inhibitor potency between PRL-1 and PRL-3 in vitro. Again we examined first the cytotoxicity of **8** toward all three cell lines with flow cytometry after treatment with different concentrations of compound **8** for 24 h (Fig 7A). Treatment with 50, 75 and 100 μM of compound **8** did not show any cytotoxic effects in the three cell lines. Next we applied dimer **8** in a concentration of 50 and 75 μM to all three cell lines in a wound healing assay. Treatment with 50 μM of **8** did not significantly increase migration behavior in the empty vector and the PRL-3 overexpressing cell lines. In contrast, treatment with 50 μM of dimer significantly decreased the number of migrating cells in the PRL-1 overexpressing cell line (see Fig 7B). Treatment with 75 μM of **8** further decreased migration in the PRL-1 overexpressing cell line, whereas it did not significantly increase the migration speed of the other two cell lines. Unlike compound **9**, compound **8** did not induce cell migration. Thus, dimer **8** is able to suppress cell migration selectively in PRL-1 overexpressing cells without affecting the empty vector and PRL-3 overexpressing cell lines, demonstrating that it acts on PRL-1.

## Discussion

In this study, we used a range of pure epi-series catechins and procyanidins to study their activity toward different PPs. We found monomeric green tea polyphenols (flavan-3-ols) such as EGCG to be only moderate inhibitors of PRL-3. In contrast, oligomers derived from (+)-catechin, the procyanidins, showed in vitro inhibitory activity in the low micromolar range toward PRLs with selectivity toward PRL-1 over PRL-2 and PRL-3. This selectivity is remarkable given the high sequence similarity of these proteins [29,33]. As there are no compounds known that are selective between the PRLs [33], procyanidins could be an interesting lead to follow up on for selective inhibitor discovery of PRLs. This conclusion is supported also by our observation of the remarkable selectivity of procyanidin C2 (**9**) over the phosphotyrosine-specific PTPs VHR, TCPTP and PTP1B. Nevertheless, the strong activity toward PP1 also needs to be taken into account. Like Erdődi and coworkers with monomeric polyphenols [27], we also observed a selectivity of the procyanidins toward PP1 over PP2A. Therefore, detailed crystal structures of PRL-1 and PP1 with procyanidins would be valuable for inhibitor development.

Testing procyanidin C2 (**9**) in cell migration assays led to the discovery that this compound alone triggers cell migration in HEK293 cells, and that the migration of PRL-1 and PRL-3-overexpressing cells was reduced when treated with **9**. This suggests that compound **9** inhibited PRL-1- and PRL-3-induced cell migration while triggering migration through unknown mechanisms. In lack of cellular substrates to study the effects of PRL inhibition, we confirmed that the enhanced cell migration phenotype in PRL-overexpressing cells depended on the PRLs by applying a PRL inhibitor (analog 3) in the wound-healing assay. This result supports further that the concentration-dependent decrease in migration speed in these cell lines caused by procyanidin C2 treatment is PRL target-specific. Also, addition of compound **9** resulted in an increase of migration in all cell lines after treatment with analog 3. Because the activity of the PRLs was inhibited, **9** could exert an effect only through unknown migration enhancing mechanisms. Thus, the activity of **9** is rather complex and it is unclear if the pathways by which the PRLs and compound **9** trigger cell migration are connected, the same or independent from each other. One point to consider as well is the membrane permeability of procyanidins. While permeation for dimeric compounds such as **8** was found up to 0.2% within 24h, for procyanidin C1, an isomer of **9**, no permeation was detected in a Caco-2 cell model [46]. On the other hand, procyanidins have been reported to interact with receptors in the cell membrane and that their activity is modulated either through direct or indirect interactions [47]. As PRL-3 is located at the inner leaflet of the cell membrane [29], procyanidin C2 (**9**) might be able to inhibit PRL-3 and thus suppress PRL-3-mediated cell migration. Nevertheless, the poor membrane permeability of trimer **9** is probably the cause for the lack of cellular selectivity of compound **9** between PRL-1 and PRL-3. Therefore, we examined procyanidin B3 (**8**), which possesses a 38-fold higher activity toward PRL-1 over PRL-3 in vitro and can enter the cells to some extent [46]. As a result compound **8** showed a clear selectivity toward the PRL-1 overexpressing cell line through significant reduction of migration speed, while the migration behavior of empty vector and PRL-3 overexpressing cell lines were not affected. Thus, this experiment proves that procyanidins exhibit selective effects between the different members of the PRL family in cells. Furthermore, the experiment with dimer **8** provides an additional proof that PRLs are targets of oligomeric catechins.

Oligomeric polyphenols were reported to have health benefits in cancer prevention [13]. Intriguingly, PRL-3 knock-down mice were shown to be about 50% less susceptible to the formation of colon cancer [48]. While in the experiments presented here higher concentrations are used than are presumably accumulated by food uptake, it is very tempting to speculate that one mechanism of cancer prevention by some polyphenols could be through the inhibition of PRL activity.

Taken together, our data indicate that both PP1 and the PRLs, in particular PRL-1 and PRL-3, could be molecular targets of procyanidins and thus contribute to the complex action of these compounds. The selectivity among the protein phosphatases tested here suggests that the action on phosphatases is specific. Furthermore, procyanidin C2 is not cytotoxic in concentrations up to 50 μM, and procyanidin B3 is either up to at least 100 μM. Together with the results reported by Erdődi and coworkers [27], these observations indicate that procyanidins and epicatechins could be interesting candidates for the inhibitor development for PRLs or PP1.

## Experimental Procedures

### Materials

Chemicals and vendors were as follows: Synthesis of Procyanidins (dimer to dodecamer) was done as previously reported [21,22]. EGCG, EGC, ECG and EC were synthesized as previously reported [17,18,49] or purchased from Sigma. (+)-Catechin and 3-(4,5-dimethylthiazol-2-yl)-2,5-diphenyltetrazolium bromide (MTT) were obtained from Sigma. 6,8-Difluoro-4-Methylumbelliferyl Phosphate (DiFMUP), propidium iodide and calcein AM were from lifescience technologies. Cell dissociation solution (10X) was purchased from amsbio. PP1c (recombinant from *E*. *coli*) was purchased from NewEngland Biolabs and PP2A (purified from human red blood cells, contains different subunits according to manufacturer) from Merck Millipore. Transwell plates (BRAND*plates*) and insert stripes for the migration assay were purchased from Brand GmbH. Black 96 well plates (96F Nunclon Delta) were purchased from Thermo Scientific. Transparent 96 well assay plates with UV transparent bottom were purchased from Corning Inc. Wound healing experiments were carried out with μ-dish 35 mm culture inserts, which were purchased from Ibidi.

### Proteins

PRL-1, PRL-2 and PRL-3 were prepared as described previously [50] with the following modifications: Plasmid vector pETM-20 was used to overexpress His-TEV-tagged fusion proteins. After lysing and purifying His-tagged proteins accordingly using a FPLC Histras HP 1 mL column, His-tags were cleaved by His-TEV protease and cleared by another round of Histrap column chromatography. VHR, PTP1B and TCPTP were prepared according to reference [44].

### Phosphatase assays

*DiFMUP assay for PRLs*: In a final reaction volume of 100 μL the PRLs (50 nM final concentration) were incubated with decreasing concentrations of the inhibitory polyphenols for 30 minutes at 25°C. The buffer conditions were 108 mM TrisCl (pH 7.5), Triton 0.05% (v/v), DTT 27.0 mM, NaCl 811 mM. After the incubation time 20 μL DiFMUP (6,8-Difluoro-4-methylumbelliferyl phosphate) was added to reach a final concentration of 21 μM for PRL-3 and 24 μM for PRL-1 and PRL-2. On a Tecan safire2 (Tecan, Salzburg, Austria) plate reader dephosphorylation of the substrate to its fluorescent product was monitored by following the emission at 358 nm with excitation at 452 nm for 20 minutes. All measurements were done in triplicates, and the experiments were repeated at least three times. The slopes of the initial phases were obtained by linear regression of the data points. The normalized reaction rates were plotted versus the log of the inhibitor concentrations and the IC_50_ values were obtained by fitting the curves using the one site competition model of GraphPad Prism (GraphPad Software, Version 5). K_M_ values were determined by measuring reaction kinetics of substrate dilution series. Initial velocities were plotted against DiFMUP concentration and the data was analyzed by Michaelis-Menten kinetics curve fitting of GraphPad Prism. A final DMSO content of 0.4% was maintained for the monomeric catechins. Due to the high water solubility of the procyanidins DMSO was not required and measurements were done in assay buffer only.

*DiFMUP assay for PTP1B*, *TCPTP*, *VHR*: In a final reaction volume of 100 μL the PTPs (50 nM final concentration) were incubated with two concentrations of procyanidin C2 (**9**) for 30 minutes at 25°C. The buffer conditions were pH 7.2, 4-/2-Hydroxyethyl)-1-piperazineethanesulfonic acid (HEPES) 25 mM, EDTA 2.5 mM, DTT 2.0 mM, NaCl 124.4 mM. After the incubation time 20 μL DiFMUP was added to reach a final concentration of 20 μM. On a Tecan safire2 (Tecan, Salzburg, Austria) plate reader dephosphorylation of the substrate to its fluorescent product was monitored by following the emission at 358 nm with excitation at 452 nm for 10 minutes. All measurements were done in triplicates, and the experiments were repeated at least three times. The slopes of the initial phases were obtained by linear regression of the data points. The normalized reaction rates were plotted versus the log of the inhibitor concentrations and the IC_50_ values were obtained by fitting the curves using the one site competition model of GraphPad Prism (GraphPad Software, Version 5). Due to the high water solubility of **9** DMSO was not required and measurements were done in assay buffer only.

*DiFMUP assay for PP1*: In a final reaction volume of 100 μL PP1c (2 mU final concentration, according to manufacturer’s instructions) were incubated with decreasing concentrations of the inhibitory polyphenols for 30 minutes at 25°C. The assay buffer contained 25 mM imidazole (pH 7.4), 1 mM dithiothreitol (DTT), and 50 mM NaCl. Bovine serum albumin (BSA) was excluded due to possible binding of the polyphenolic compounds. After the incubation time 20 μL DiFMUP (6,8-Difluoro-4-methylumbelliferyl phosphate) was added to reach a final concentration of 100 μM. Monitoring of the fluorescent dephosphorylation product was monitored and data analyzed as described above.

*DiFMUP assay for PP2A*: In a final reaction volume of 100 μL PP2A (0.05 U final concentration, according to manufacturer’s instructions) were incubated with decreasing concentrations of the inhibitory polyphenols for 30 minutes at 25°C. The assay buffer contained 50 mM TrisCl (pH 7.5), 250 mM NaCl, 0.1 mM EDTA, 1 mM MnCl_2_, and 2 mM dithiothreitol (DTT). Bovine serum albumin (BSA) was excluded due to possible binding of the polyphenolic compounds. After the incubation time 20 μL DiFMUP (6,8-Difluoro-4-methylumbelliferyl phosphate) was added to reach a final concentration of 100 μM. Monitoring of the fluorescent dephosphorylation product was monitored and data analyzed as described above.

### Cell lines

For HEK293 empty vector and stably overexpressing PRL-1 and -3 cell lines see refs [50].

### Flow cytometry

Flow cytometry was used to determine the ratio of living to dead cells compared to the control. HEK293 empty vector and PRL-3 overexpressing cells were seeded in a transparent 6-well plate with 400,000 cells/well in DMEM medium without Phenol red, supplemented with 10% FBS, 1% glutamine and 0.2% hygromycin and blasticidin. For induction of PRL-3 expression, 0.1% tetracycline was added. After 24 hours incubation at 37°C, procyanidins **8** or **9** dissolved in growth medium and growth medium as a control were added to the respective wells in the indicated final concentrations and incubated for another 24 hours. Then, the medium was removed and collected in Eppendorf tubes, centrifuged for 5 min at 500 rpm and the supernatant removed. Then 1 mL of PBS (10%) was added to each well and the cells detached with a cell scraper. The cell suspension was then transferred to the Eppendorf tubes containing the fraction of dead cells and centrifuged for 5 in at 500 rpm. Again 1 mL of PBS (10%) was added to the cell pellet, the cells were re-suspended and centrifuged again. The supernatant was removed, the cells re-suspended in 400 μl PBS (10%) and 0.8 μL of propidium iodide (1 mg/mL) added. Then the cell suspension was incubated for 5 min on ice. After thorough mixing the cell suspension was injected into a LSR- Fortessa Bench Top Analyzer (Beckton Dickinson) and the number of living and dead cells counted. As a threshold, 10,000 events were set for the number of living cells. The number of living cells was divided by the number of total cells counted and given as percentage of cell survival.

### Wound healing assay

Wound healing experiments were carried out with μ-dish 35mm culture inserts. 8 × 10^5^ cells/mL of the respective HEK293 stable cell lines were seeded into each of the two insert wells and allowed to settle for 40 h in the presence of tetracycline. Then the inserts were gently removed and growth medium supplemented with 7.5% FBS and 0.1% tetracycline was added as well as procyanidin C2 (**9**) or procyanidin B3 (**8**) at a final concentration of 25, 50 or 75 μM. For the experiment including blocking of PRLs with analog 3, the latter one was added at a final concentration of 35 μM, followed by addition of procyanidin C2 (**9**) at either 25 or 50 μM final concentration. Negative controls contained growth medium with FBS only. Pictures of the wound gap were taken at 0 h and 7 h time points. A Zeiss Cellobserver HS microscope was used with 10 × magnitude. For data analysis, in each experiment, the distances between both cell fronts at time point 0 h and 7 h were measured by taking 20 different measuring points. Then all the measurements from different replicates (4) for each experiment were combined and analyzed by t-test using Welsh correction in GraphPad Prism. Data are shown with 99% confidence intervals.

### Transwell assay

HEK293 empty vector, PRL-1 and PRL-3 overexpressing cells were kept in selection medium (DMEM medium without Phenol red, supplemented with 10% FBS, 1% glutamine and 0.2% hygromycin and blasticidin) for one week until full confluency. Then, 0.1% tetracycline (1 mg/ml stock solution in ethanol) was added for induction of PRL-1 and PRL-3 expression in HEK293 PRL-1 and HEK PRL-3 wt 24 h prior plating on transwell inserts. For comparison, HEK293 empty vector was also treated with tetracycline. After 24 h, the medium was removed, cells were washed with PBS and starvation medium supplemented with 0.1% tetracycline was added. Cells were incubated for 6 h at 37°C. Then, cells were harvested by trypsinization, washed with PBS buffer, and re-suspended in starvation medium supplemented with 0.1% tetracycline. In 12 well transwell permeable inserts with 8 μm pores 100 μL of cell suspension were plated with a total count of 100,000 cells/well. Procyanidin C2 was added at a final concentration of 25 or 50 μM. For each cell line with and without drug, four replicates were used per experiment. The receiver wells were filled with 750 μL of growth medium containing 5% FBS and cells were incubated for 16 hours at 37°C. Then, the medium in the inserts and receiver wells was removed and inserts carefully washed in PBS. 700 μL of 1X cell dissociation solution supplemented with 1.2 μg/mL calcein AM was added to each receiver well thereby wetting the inserts. The plate was incubated for 1 h at 37°C. Then, 3x100 μL were taken from each well and transferred to a black assay plates and the relative concentration of the fluorescent product formed in the cells was determined by following the emission at 520 nm with excitation at 452 nm. For data analysis, the average of the absorption units obtained for each concentration and cell line (12 wells) was normalized to the empty vector control. Then the normalized absorption units from different replicates for each experimental design were combined, and analyzed by t-test using Welsh correction in GraphPad Prism. Data are shown with 99% confidence intervals.
